# Transcranial Alternating Current Stimulation Modulates Risky Decision Making in a Frequency-Controlled Experiment

**DOI:** 10.1523/ENEURO.0136-17.2017

**Published:** 2017-12-11

**Authors:** Zachary Yaple, Mario Martinez-Saito, Bhuvanesh Awasthi, Matteo Feurra, Anna Shestakova, Vasily Klucharev

**Affiliations:** 1 Centre for Cognition and Decision Making, National Research University Higher School of Economics, 20 Myasnitskaya Ulitsa, Moscow 109316, Russian Federation; 2 School of Psychology, National Research University Higher School of Economics, Armyanskiy per.4,c2, Moscow 109316, Russian Federation

**Keywords:** 20-Hz stimulation, frontal hemisphere, reward, risky decision making, task switching, transcranial alternating current stimulation

## Abstract

In this study, we investigated the effect of transcranial alternating current stimulation (tACS) on voluntary risky decision making and executive control in humans. Stimulation was delivered online at 5 Hz (θ), 10 Hz (α), 20 Hz (β), and 40 Hz (γ) on the left and right frontal area while participants performed a modified risky decision-making task. This task allowed participants to voluntarily select between risky and certain decisions associated with potential gains or losses, while simultaneously measuring the cognitive control component (voluntary switching) of decision making. The purpose of this experimental design was to test whether voluntary risky decision making and executive control can be modulated with tACS in a frequency-specific manner. Our results revealed a robust effect of a 20-Hz stimulation over the left prefrontal area that significantly increased voluntary risky decision making, which may suggest a possible link between risky decision making and reward processing, underlined by β-oscillatory activity.

## Significance Statement

This is the first study that shows a frequency-specific effect on risky decision making demonstrated by online 20-Hz transcranial alternating current stimulation (tACS) applied to left dorsolateral prefrontal cortex (PFC). Our results suggest that left frontal 20-Hz tACS specifically modulates risky decision making, perhaps by entraining endogenous β-activity underlying a frontal-striatal network associated with gain anticipation.

## Introduction

Much research has been conducted on the neurobiological mechanisms of risky decision making demonstrating a large neural network comprised of the ventral striatum, amygdala, insula, cingulate, and prefrontal cortices (PFCs; Knutson et al., 2001a,b; O'Doherty et al., 2001; Kuhnen and Knutson, 2005; Rao et al., 2008; Fujiwara et al., 2009; Mohr et al., 2010; Kohls et al., 2013). In particular, the PFC plays an important role in voluntary risky decision making. For instance, Rao et al. (2008) demonstrated a link between the PFC and voluntary decisions to accept greater risk. They suggested that the PFC mediates the active volitional control or agency of the risk taker by means of an executive control component.

The PFC also plays a prominent role in executive control (Derrfuss et al., 2005; Swick et al., 2011; Kim et al., 2012; Rottschy et al., 2012; also, see Owen et al., 2005, for fMRI meta-analyses on executive functions), which in turn comprises of three separate, independent components; working memory updating, inhibition, and set shifting/task switching (Miyake et al., 2000; Diamond, 2013). Risky decision making and executive control have been thoroughly investigated. Inspired by Kahneman’s dual process theory, that irrational decision making increases when cognitive resources become depleted (Kahneman, 2003; Kahneman and Frederick, 2007; Kahneman, 2011), some have tested the influence of executive control on risky decision making by administering the n-back task, a popular working memory task, in parallel with various risky decision-making tasks (Whitney, Rinehart and Hinson, 2008; Starcke et al., 2011; Farrell et al., 2012; Pabst et al., 2013; Gathmann et al., 2014a,b). Likewise, many have examined inhibitory processes and risky decision making by employing the Go No-Go (Verdejo-García et al., 2007; Yeomans and Brace, 2015; Ba et al., 2016; Welsh et al., 2017). However, to date few have examined the link between set-switching and risky decision making (Verdejo-García et al., 2007; Fröber and Dreisbach, 2016); therefore, we proposed to investigate this link by using brain stimulation of the PFC.

θ-Related activity (4–8 Hz) has been inferred to reflect aspects of risky decision making and executive control. While numerous accounts have associated θ-band oscillations with executive control functions (e.g., working memory, set-switching, conflict monitoring, error detection; Jensen and Tesche, 2002; Sauseng et al., 2006; Cunillera et al., 2012; Cavanagh and Frank, 2014), a recent EEG study reported fronto-central θ-oscillations inferred to reflect an action monitoring system that compares potential outcomes of high- and low-risk options (Zhang et al., 2014). Furthermore, θ-band transcranial alternating current stimulation (tACS) applied on the left PFC was demonstrated to increase risky decision making (Sela et al., 2012). This stimulation technique allegedly entrains ongoing electrophysiological oscillatory activity (Veniero et al., 2015; Vosskuhl et al., 2015; Thut et al., 2011; Helfrich et al., 2014a), suggesting that theta tACS entrains frontal-central theta oscillations. However, a disadvantage to this study is that frequency specificity could not be assessed since the authors did not control for other stimulation frequencies. In other words, the increase in risky decision making may have been driven by the stimulation alone and not necessarily by theta stimulation (for further details, see Feurra et al., 2012).

For this study, we tested whether voluntary risky decision making under varied levels of executive control can be modulated by applying online tACS at various frequencies (sham, 5, 10, 20, and 40 Hz) to the left and right frontal hemispheres. To isolate these components of decision making, we adopted and modified a task-switching paradigm that allows participants to choose between risky and safe (certain) decisions depending on the decision to switch or repeat between task sets (Arrington and Logan, 2004, 2005; Weaver and Arrington, 2013; Arrington et al., 2014; Orr and Banich, 2014; Poljac and Yeung, 2014; Fröber and Dreisbach, 2016). Although relatively new for cognitive neuroscience (Orr and Banich, 2014; Poljac and Yeung, 2014), the voluntary task-switching paradigm is well established within the cognitive psychological literature (Arrington and Logan, 2004, 2005; Weaver and Arrington, 2013; Fröber and Dreisbach, 2016). However, unlike typical executive tasks, in which participants are rated on response time and accuracy (e.g., N back, Go Go-No task, Eriksen Flanker task, Wisconsin Card Sorting task), the voluntary task-switching paradigm investigates voluntary executive control by considering choice as a dependent variable. By combining the voluntary task-switching paradigm with two-choice financial decision-making task between lotteries involving risk, it is possible to measure how much executive control participants are willing to exert under the condition of risk. The advantage of this task design was the possibility to measure voluntary executive control and voluntary risky decision making within a single response, thus allowing us to test whether tACS can modulate voluntary risky decision making under varied levels of voluntary executive control. Given that voluntary, but not involuntary, risky decision making yields frontal-ventral striatum activity (Rao et al., 2008), we hypothesize that θ-band tACS should modulate voluntary risky decision making under high levels of executive control.

## Materials and Methods

### Participants

Thirty-four healthy right-handed participants (21 females; mean age 21; age range 18–26 years; SD = 2.54) with normal or corrected to normal vision and with no neurologic disorders participated in the study. All participants provided a written consent approved by a local ethics committee in accordance with the Declaration of Helsinki. All participants were screened for psychological/psychiatric disorders and none of them reported use of drugs or alcohol in the days preceding the experiment. Participants were divided into two groups: those who received stimulation on the left frontal area (*n* = 17; 10 females; mean age 20.52; age range 18–25 years; SD = 2.52) and those who received stimulation on the right frontal area (*n* = 17; 11 females; mean age 21.17; age range 18–26 years; SD = 2.78).

### Stimuli and procedure

Participants performed a novel neuroeconomic risky decision-making task that combines binary lotteries with equal expected value (Selten et al., 1999; Engelmann and Tamir, 2009; Harrison et al., 2013), and the voluntary task-switching paradigm (Arrington and Logan, 2004, 2005) that allows participants to select between risky or certain decisions by switching or repeating task sets between trials. Each trial began with a centered fixation cross which remained between 500 and 1000 ms followed by the stimuli screen, composed of a randomly selected single digit (1, 2, 3, 4, 6, 7, 8, or 9) centered on the screen until the participant responded. For each trial participants had to select one of the two games: odd/even game (participants indicated whether the digit was odd or even) or higher/lower game (participants indicated whether the digit was higher or lower than 5) by pressing one of the corresponding buttons (odd, even, high, low). Using a randomized Latin-square blocked design, the instruction varied across blocks as described below.

**Figure 1.**
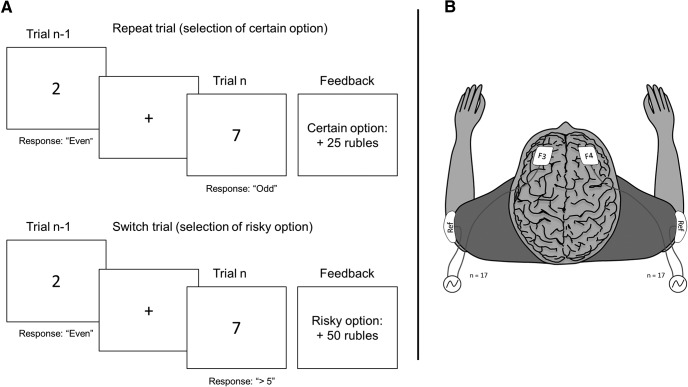
***A***, Rewarded voluntary switch task, a combined risky decision-making and task-switching paradigm. In trial N, subjects may select the certain decision (25 RUB with a probability of 100%) or the risky decision (50 RUB or 0 RUB with a probability of 50%) depending on decision to switch or repeat task sets from trial N-1. Figure represents trial in the reward switch = risk block. ***B***, tACS montage. Active electrodes were placed on F3 and F4 electrode, representing left and right frontal area. Placement of the reference electrode was the ipsilateral deltoid for F3 and F4.

In the basic version of the task (Fig. 1*A*, “switch = risk” blocks) participants were instructed that if they chose to repeat the same game in successive trials they would make a certain decision, e.g., they select “odd” button for the digit 3 on trial N-1, and then “even” button for the digit 8 on trial N, repeating the odd/even game. If the participant decided to alternate between game types, participants made a risky decision, e.g., they select “odd” button for the digit 3 on trial N-1, and then “high” button for the digit 8 on trial N, switching to the higher/lower game. Across half of the blocks these instructions were counterbalanced such that switching between games led to the certain decision and repeating the same game would yield the risky decision. In the Results section these block instructions are referred to as switch = risk blocks and “repeat = risk” blocks. In other words, to select a risky decision, participants had to switch between games (switch = risk blocks), while in the other blocks (repeat = risk blocks), participants had to repeat the same game.

Since gain-framed and loss-framed decisions differentially affect risk preferences (Tversky and Kahneman, 1985), the experiment was also divided into gain and loss blocks. In gain blocks, certain decisions were defined and instructed as “100% probability that you would receive 25 Russian rubles (RUB)”, while risky decisions were defined and instructed as “50% probability that you would receive 50 RUB” (or alternatively 0 RUB). In loss blocks, the certain decision indicated “100% probability that you would lose 25 RUB” while risky decisions indicated “50% probability that you would lose 50 RUB” (alternatively 0 RUB). For each response that determined the game they selected, a feedback screen displayed for 1000 ms indicated the amount of money gained or lost for that particular trial. If response time exceeded 4000 ms or participants responded erroneously, feedback for that particular trial displayed negative feedback (e.g., 0 RUB for gain block, −50 RUB for loss blocks).

Similarly to the voluntary switching task, response buttons were counterbalanced across participants (Arrington and Logan, 2004). Block condition were counterbalanced in random order. Presentation of stimuli and recording of responses were controlled by E-Prime 2.0 software. All text was displayed in black font on a gray scale background and all participants were instructed to use two hands to respond. Due to the difficulty of the task and to avoid learning effects, participants received two rounds of training, which consisted of eight blocks of 10 trials, resulting in 80 trials in total. If accuracy was below 95% additional training sessions were given. This learning phase was reflected in the actual experiment in which accuracy for all participants throughout the task was above 92%. After training, participants received 20 blocks of 20 trials each. At the end of the experiment participants were shown the total cumulative feedback on the computer screen. Participants received 500 RUB for participation (500 RUB ≈ 7 United States dollar) and an additional bonus, between −300 and +300 RUB, based on the feedback outcomes of six randomly selected trials to maintain an equal motivation for risky decision making across blocks (Krajbich et al., 2012).

### tACS procedure

By using the international electroencephalography 10-20 system, tACS was applied on the left or right frontal areas by placing a 7 × 5-cm saline-soaked electrode on F3 or F4 locations (Fig. 1*B*). For both location sites, a reference electrode was placed on the ipsilateral deltoid to the target electrode (Im et al., 2012; Bai et al., 2014). The order of stimuli was randomized across 20 blocks. Standard protocols were employed as in previous frequency-controlled tACS experiments on motor and cognitive tasks (sham, 5, 10, 20, and 40 Hz; Feurra et al., 2011, 2016; Santarnecchi et al., 2013, 2016), accounting for mean center frequencies (Klimesch, 2012). Furthermore, tACS set at a fixed frequency has been shown to entrain individualized α-oscillations converging to a 10-Hz stimulation (Helfrich et al., 2014a). Therefore, we contend that these frequency stimulations suffice to entrain endogenous neural oscillations within standard θ, α, β, and γ ranges, respective of individual frequency ranges.

Stimulation was delivered online during task performance, with exception to sham stimulation, which lasted for 30 s. To implement a sham stimulation, instead of using a fixed frequency that may bias a single stimulation protocol over another, we applied sinusoidal low-frequency transcranial random noise stimulation (tRNS) between 0.1 and 100 Hz for 30 s. This sham stimulation protocol was necessary in the current experiment due to the unconventional use of multiple stimulation protocols reflecting the harmonics of mean center frequencies (Klimesch, 2012). Furthermore, it is important to emphasize that low-frequency tRNS was applied only for a short duration, compared to all other protocols that were applied throughout the entire block; sham stimulation was delivered for 30 s with 10-s fade-in/fade-out, while all other stimulation protocols lasted between 5 and 10 min. Moreover, low-frequency tRNS has been shown not to affect cortical excitability (Paulus, 2011). Stimulation current was set at 1 mA (500 mA peak-to-peak). The maximum current density at the stimulation electrode was ∼14 μA/cm^2^. The wave form of the stimulation was sinusoidal, and there was no direct current offset. The low intensity of stimulation was used to avoid a perception of flickering lights (Paulus, 2010). Stimulation was delivered using a battery-operated stimulator system (BrainStim, EMS Medical). Impedance was kept below 10 kΩ. All protocols began one minute before each block. Due to abundant evidence that tACS affects physiologic activity during stimulation (Antal et al., 2008; Helfrich et al., 2014a,b; Strüber et al., 2015), breaks of 5 min were given after each set of four blocks. In total, stimulation lasted ∼40 min.

### Statistical analysis

Analysis was performed using R software (R Core Team, 2016) with the software package lme4 (Bates et al., 2014) and lmertest (Kuznetsova et al., 2016). Two separate logistic regression mixed models (Generalized Linear Mixed Model) on the raw data were performed on the following variables: (1) selection of risky decisions and (2) selection of switches between trials. Each model included the following categorical predictors: valence (gain, loss blocks), switch condition (switch = risk blocks and repeat = risk), frequency of stimulation (sham, 5, 10, 20, and 40 Hz) with sham as a reference variable, and hemisphere of stimulation (left, right). Before analysis error trials and trials exceeding response time of four seconds were omitted. Wald tests (Kuznetsova et al., 2016) were performed on all levels up to two interactions. To account for possible group differences, sham stimulation was used as a reference variable for each effect associated with frequency. In the logistic regression model participants, valence, switch condition, and frequency of stimulation were modeled with random effects, while hemisphere of stimulation (a between-subjects factor) was modeled with fixed effects. The R command lme4 function is as follows: glmer(Risk ∼ (Frequency + Valence + Hemi) ^ 2 + (1 + Frequency + Condition + Valence:Condition | Subject), family = “binomial”, data = D, control = glmerControl (optimizer=“bobyqa”, optCtrl = list (maxfun = 2e5))). Significance for the regression coefficients was corrected for false positives by using Holm-Bonferroni procedure.

For the following analyses we used SPSS software version 20 (IBM Corp, 2011). A mixed ANOVA was performed on the mean response time of the following variables: valence (gain, loss blocks), frequency of stimulation (sham, 5, 10, 20, and 40 Hz), switch condition (switch = risk blocks and repeat = risk blocks), and hemisphere of stimulation (left- and right-stimulated group), in which switch condition, valence, and frequency of stimulation were within-participants factors and hemisphere of stimulation was treated as between participants factor. Sphericity was not violated across any of these effects (all *p* > 0.05). To assess whether participants selected more risky decisions than chance level, a one-sample *t* test was performed.

## Results

**Figure 2.**
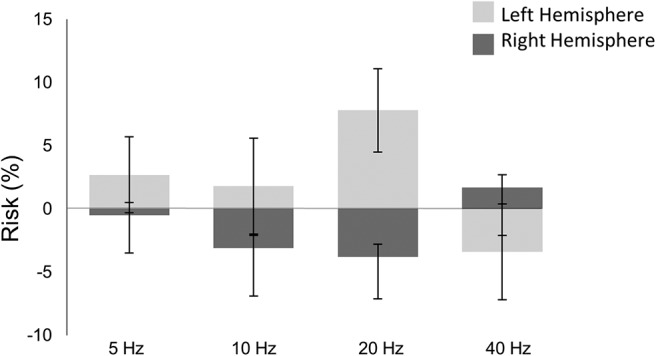
Mean percentage of risky decisions for each tACS condition with respect to sham; 20-Hz stimulation of the left frontal area increased selection of voluntary risky decisions. Error bars correspond to SEM.

Figure 2 displays the percentage of risky decisions in all stimulation conditions. The logistic regression mixed model for risky decision making revealed an increase in risky decision making during 20 Hz of stimulation particularly when stimulating the left PFC (β = 0.989; *p* = 0.00194; *p’* = 0.043). The effects of other tACS frequencies on risky decision making did not survive Holm-Bonferroni correction for multiple comparisons (Table 1). The frequency- and hemisphere-specific effect of a 20-Hz stimulation was confirmed by a nonsignificant main effect of hemisphere of stimulation (β = 0.072, *p* = 0.885; *p’* > 0.999). Figure 2 displays means and standard error for each of the comparisons with regards to the frequency of stimulation × hemisphere of stimulation interaction effect.

**Table 1.** Results of the logistic regression for risky decision making and for voluntary switching

Logistic regression model of risky decision making (sham as reference variable)						Logistic regression model of voluntary switching (sham as reference variable)					
	β	SE	*z* value	*p* value	*p’*		β	SE	*z* value	*p* value	*p’*
5 Hz (sham)	0.248	0.213	1.163	0.244	>0.99	5 Hz (sham)	−0.196	0.170	−1.153	0.249	>0.99
10 Hz (sham)	0.172	0.287	0.600	0.548	>0.99	10 Hz (sham)	−0.034	0.148	−0.232	0.816	>0.99
20 Hz (sham)	0.624	0.251	2.486	0.012	0.265	20 Hz (sham)	−0.054	0.138	−0.394	0.693	>0.99
40 Hz (sham)	−0.204	0.283	−0.720	0.471	>0.99	40 Hz (sham)	−0.225	0.162	−1.394	0.163	>0.99
Switch Cond	−0.496	0.234	−2.118	0.034	0.581	**Switch Cond**	**2.005**	**0.103**	**19.361**	**<2×10^−16^**	**<0.001**
Hemisphere (L-R)^a^	0.072	0.501	0.144	0.885	>0.99	Hemisphere (L-R)^a^	−0.402	0.198	−2.031	0.042	0.714
Valence (gain-loss)	0.693	0.476	1.455	0.145	>0.99	Valence (gain-loss)	−0.110	0.103	−1.068	0.285	>0.99
5 Hz* (gain-loss)	−0.156	0.132	−1.183	0.236	>0.99	5 Hz* (gain-loss)	0.027	0.121	0.229	0.818	>0.99
10 Hz* (gain-loss)	−0.305	0.133	−2.284	0.022	0.425	10 Hz* (gain-loss)	0.258	0.119	2.163	0.030	0.540
20 Hz* (gain-loss)	−0.166	0.133	−1.247	0.212	>0.99	20 Hz* (gain-loss)	0.058	0.120	0.486	0.626	>0.99
40 Hz* (gain-loss)	−0.233	0.131	−1.768	0.076	>0.99	40 Hz* (gain-loss)	0.215	0.120	1.790	0.073	>0.99
5 Hz* Switch Cond	−0.125	0.132	−0.945	0.344	>0.99	5 Hz* Switch Cond	0.083	0.123	0.676	0.499	>0.99
10 Hz* Switch Cond	0.076	0.133	0.573	0.566	>0.99	10 Hz* Switch Cond	−0.174	0.120	−1.450	0.147	>0.99
20 Hz* Switch Cond	0.003	0.133	0.026	0.979	>0.99	20 Hz* Switch Cond	0.113	0.122	0.930	0.352	>0.99
40 Hz* Switch Cond	−0.026	0.131	−0.205	0.837	>0.99	40 Hz* Switch Cond	−0.126	0.122	−1.032	0.302	>0.99
5 Hz* (L-R)^a^	0.202	0.270	0.747	0.455	>0.99	5 Hz* (L-R)^a^	−0.185	0.202	−0.915	0.360	>0.99
10 Hz* (L-R)^a^	0.418	0.384	1.091	0.275	>0.99	10 Hz* (L-R)^a^	−0.153	0.166	−0.919	0.358	>0.99
20 Hz* (L-R)^a^	**0.989**	**0.319**	**3.099**	**0.001**	**0.043**	20 Hz* (L-R)^a^	−0.027	0.147	−0.190	0.849	>0.99
40 Hz* (L-R)^a^	−0.276	0.380	−0.726	0.467	>0.99	40 Hz* (L-R)^a^	−0.353	0.187	−1.884	0.059	0.944
Switch Cond* (gain-loss)	−0.385	0.154	−2.494	0.012	0.265	Switch Cond* (gain-loss)	**−0.311**	**0.076**	**−4.082**	**4.47×10^−5^**	**0.001**
Switch Cond* (L-R)^a^	−0.284	0.305	−0.931	0.351	>0.99	Switch Cond* (L-R)^a^	**0.908**	**0.076**	**11.799**	**<2×10^−16^**	**<0.001**
(gain-loss)* (L-R)^a^	1.442	0.657	2.195	0.028	0.507	(gain-loss)* (L-R)^a^	−0.215	0.076	−2.822	0.004	0.076

Each frequency is referenced to sham and for hemisphere, left-stimulated group (L) is respect to right frontal stimulated group (R). β = β coefficient; SE = SEM; *z* value based on Wald test; *p’* indicates adjusted *p* values by Holm-Bonferroni correction; bold text indicates significant *p* values; Switch Cond: switch = risk blocks minus repeat = risk blocks; sham as reference variable for frequency of stimulation; a = includes predictor modeled as fixed effects.

In addition, separate logistic regression models were performed using sham as a reference for each stimulation group (Table 2). The model for the left-stimulated group (Table 2) revealed a statistical significant increase in risky decision making from a 20-Hz stimulation (β = 0.610, *p* = 0.001; *p’* > 0.021). Follow-up analysis using 20 Hz as a reference (Table 3) revealed that 20 Hz of stimulation applied to the left hemisphere increased risky decision making with respect to sham (β = −0.989, *p* = 0.001; *p’* > 0.021) and 40-Hz stimulation (β = −1.265, *p* < 0.001; *p’* > 0.015). When separately testing hemisphere stimulation groups with 20 Hz as the reference variable, 20 Hz increased risky decision making compared to sham for the left-stimulated group (β = −0.610, *p* = 0.001; *p’* > 0.021; Table 4). No effects were found for the right-stimulated group (Table 4).

**Table 2.** Logistic regression model of risky decision making for each group with sham as a reference variable

Logistic regression model of risky decision making for left hemisphere group (sham as reference variable)						Logistic regression model of risky decision making for right hemisphere group (sham as reference variable)						
	β	SE	*z* value	*p* value	*p’*		β	SE	*z* value	*p* value	*p’*	
5 Hz (sham)	0.160	0.241	0.661	0.508	>0.99	5 Hz (sham)	0.130	0.246	0.529	0.596	>0.99	
10 Hz (sham)	0.309	0.283	1.091	0.275	>0.99	10 Hz (sham)	−0.383	0.340	−1.127	0.259	>0.99	
**20 Hz (sham)**	**0.610**	**0.239**	**2.545**	**0.001**	**0.021**	20 Hz (sham)	−0.352	0.326	−1.079	0.280	>0.99	
40 Hz (sham)	−0.065	0.299	−0.219	0.826	>0.99	40 Hz (sham)	−0.114	0.318	−0.359	0.719	>0.99	
Switch Cond	−0.361	0.319	−1.131	0.258	>0.99	Switch Cond	−0.371	0.199	−1.865	0.062	0.868	
Valence (gain-loss)	0.672	0.341	1.970	0.048	0.672	Valence (gain-loss)	−0.844	0.661	−1.277	0.201	>0.99	
5 Hz* (gain-loss)	0.119	0.193	0.617	0.537	>0.99	5 Hz* (gain-loss)	−0.350	0.184	−1.900	0.057	0.855	
10 Hz* (gain-loss)	−0.373	0.192	−1.937	0.052	0.676	10 Hz* (gain-loss)	−0.268	0.187	−1.437	0.150	>0.99	
20 Hz* (gain-loss)	0.001	0.198	0.006	0.995	>0.99	20 Hz* (gain-loss)	−0.273	0.183	−1.489	0.136	>0.99	
40 Hz* (gain-loss)	−0.207	0.190	−1.093	0.274	>0.99	40 Hz* (gain-loss)	−0.256	0.186	−1.379	0.167	>0.99	
5 Hz* Switch Cond	−0.217	0.193	−1.125	0.260	>0.99	5 Hz* Switch Cond	−0.040	0.183	−0.223	0.823	>0.99	
10 Hz* Switch Cond	−0.373	0.192	−1.937	0.052	0.672	10 Hz* Switch Cond	0.231	0.183	1.257	0.208	>0.99	
20 Hz* Switch Cond	0.001	0.198	0.006	0.995	>0.99	20 Hz* Switch Cond	0.085	0.180	0.474	0.635	>0.99	
40 Hz* Switch Cond	−0.207	0.190	−1.093	0.274	>0.99	40 Hz* Switch Cond	0.242	0.182	1.327	0.184	>0.99	
Switch Cond* (gain-loss)	−0.476	0.270	−1.758	0.078	0.858	Switch Cond* (gain-loss)	−0.281	0.209	−1.340	0.180	>0.99	

Results display β coefficients (β) with SE, *z* score, original *p* value, and corrected *p* value (*p’*) for the following predictors: frequency (5, 10, 20, and 40 Hz and sham), switch condition (trials in which switch = risk minus trials in which repeat = risk), and valence (gain minus loss trials). All predictors were modeled with random effects. Bold text indicates significance after Holm-Bonferonni correction.

**Table 3.** Logistic regression model of risky decision making with 20 Hz as a reference variable

	β	SE	*z* value	*p* value	*p’*
Sham (20 Hz)	−0.624	0.251	−2.482	0.013	0.216
5 Hz (20 Hz)	−0.375	0.240	−1.565	0.117	>0.99
10 Hz (20 Hz)	−0.451	0.256	−1.764	0.077	0.975
40 Hz (20 Hz)	−0.828	0.291	−2.844	0.004	0.080
Switch Cond	−0.493	0.236	−2.087	0.036	0.504
Hemisphere (L-R)^a^	1.061	0.423	2.504	0.012	0.216
Valence (gain-loss)	0.526	0.476	1.104	0.269	>0.99
Sham* (gain-loss)	0.166	0.133	1.247	0.212	>0.99
5 Hz* (gain-loss)	0.009	0.134	0.072	0.942	>0.99
10 Hz* (gain-loss)	−0.138	0.134	−1.031	0.302	>0.99
40 Hz* (gain-loss)	−0.066	0.133	−0.499	0.617	>0.99
Sham* Switch Cond	−0.003	0.133	−0.026	0.979	>0.99
5 Hz* Switch Cond	−0.128	0.134	−0.959	0.337	>0.99
10 Hz* Switch Cond	0.073	0.134	0.544	0.586	>0.99
40 Hz* Switch Cond	−0.030	0.133	−0.228	0.819	>0.99
Sham* (L-R)^a^	**−0.989**	**0.319**	**−3.097**	**0.001**	**0.021**
5 Hz* (L-R)^a^	−0.786	0.304	−2.588	0.009	0.171
10 Hz* (L-R)^a^	−0.570	0.321	−1.774	0.075	0.975
40 Hz* (L-R)^a^	**−1.265**	**0.374**	**−3.380**	**<0.001**	**0.015**
Switch Cond* (gain-loss)	−0.385	0.154	−2.495	0.012	0.216
Switch Cond* (L-R)^a^	−0.284	0.305	−0.931	0.351	>0.99
(gain-loss)* (L-R)^a^	1.442	0.656	2.198	0.027	0.405

Results display β coefficients (β) with SE, *z* score, original *p* value, and corrected *p* value (*p’*) for the following predictors: frequency (5, 10, 20, and 40 Hz and sham), switch condition (trials in which switch = risk minus trials in which repeat = risk), valence (gain minus loss trials), and hemisphere of stimulation (left group minus right group). Frequency, switch condition, and valence were modeled as random effects; hemisphere of stimulation was modeled with fixed effects due to a between-subjects factor; 20 Hz as reference variable for frequency of stimulation; ^a^ = includes predictor modeled as fixed effects; bold text indicates significance after Holm-Bonferonni correction.

**Table 4.** Logistic regression model of risky decision making for each group with 20 Hz as a reference variable

Logistic regression model of risky decision making for left hemisphere group (20 Hz as reference variable)						Logistic regression model of risky decision making for right hemisphere group (20 Hz as reference variable)					
	β	SE	*z* value	*p* value	*p’*		β	SE	*z* value	*p* value	*p’*
Sham (20 Hz)	−0.610	0.239	−2.550	0.001	0.021	Sham (20 Hz)	0.352	0.326	1.079	0.280	>0.99
5 Hz (20 Hz)	−0.450	0.264	−1.703	0.088	0.968	5 Hz (20 Hz)	0.483	0.276	1.747	0.080	>0.99
10 Hz (20 Hz)	−0.301	0.355	−0.850	0.395	>0.99	10 Hz (20 Hz)	−0.031	0.201	−0.155	0.876	>0.99
40 Hz (20 Hz)	−0.676	0.397	−1.701	0.089	0.968	40 Hz (20 Hz)	0.237	0.223	1.066	0.286	>0.99
Switch Cond	−0.468	0.325	−1.441	0.149	>0.99	Switch Cond	−0.286	0.193	−1.475	0.140	>0.99
Valence (gain-loss)	0.673	0.346	1.946	0.051	0.714	Valence (gain-loss)	−1.117	0.662	−1.688	0.091	>0.99
Sham* (gain-loss)	−0.001	0.198	−0.006	0.995	>0.99	Sham* (gain-loss)	0.273	0.183	1.490	0.136	>0.99
5 Hz* (gain-loss)	0.118	0.200	0.590	0.554	>0.99	5 Hz* (gain-loss)	−0.076	0.183	−0.419	0.675	>0.99
10 Hz* (gain-loss)	−0.374	0.199	−1.881	0.060	0.780	10 Hz* (gain-loss)	0.004	0.184	0.027	0.978	>0.99
40 Hz* (gain-loss)	−0.209	0.197	−1.060	0.289	>0.99	40 Hz* (gain-loss)	0.016	0.184	0.091	0.927	>0.99
Sham* Switch Cond	0.107	0.200	0.536	0.591	>0.99	Sham* Switch Cond	−0.085	0.179	−0.474	0.635	>0.99
5 Hz* Switch Cond	−0.110	0.202	−0.545	0.585	>0.99	5 Hz* Switch Cond	−0.126	0.180	−0.698	0.485	>0.99
10 Hz* Switch Cond	0.015	0.203	0.077	0.938	>0.99	10 Hz* Switch Cond	0.145	0.180	0.809	0.418	>0.99
40 Hz* Switch Cond	−0.202	0.200	−1.014	0.310	>0.99	40 Hz* Switch Cond	0.157	0.179	0.875	0.381	>0.99
Switch Cond* (gain-loss)	−0.476	0.270	−1.759	0.078	0.936	Switch Cond* (gain-loss)	−0.281	0.209	−1.340	0.180	>0.99

Results display β coefficients (β) with SE, *z* score, original *p* value, and corrected *p* value (*p’*) for the following predictors: frequency (5, 10, 20, and 40 Hz and sham), switch condition (trials in which switch = risk minus trials in which repeat = risk), and valence (gain minus loss trials). All predictors were modeled with random effects.

After Holm-Bonferroni correction, the logistic regression mixed model yielded no significant effects of tACS on voluntary switching, yet revealed a main effect of switch condition (β = 2.005; *p* ≤ 2 × 10^−16^; *p’* < 0.001), an interaction effect of switch condition × valence (β = −0.311; *p* = 4.47 × 10^−5^; *p’* = 0.001), and an interaction effect of switch condition × hemisphere of stimulation (β = 0.908; *p* ≤ 2 × 10^−16^; *p’* < 0.001). These effects indicate an increase in voluntary switching in the switch = risk blocks compared to repeat = risk blocks, especially for loss blocks; perhaps reflecting an influence of executive control on the framing bias. The interaction effect of switch condition and Hemisphere may demonstrate an increase in voluntary switching during switch = risk blocks compared to repeat = risk blocks from the left-stimulated group, yet should be interpreted with caution since Hemisphere of stimulation was modeled with fixed effects (see Discussion for details). Analysis of response times revealed that participants responded more slowly in trials in which switching between tasks led to risk (μ = 1112.43 ms) compared to trials in which repeating led to risk (μ = 988.98 ms; *F*_(1,32)_ = 17.455; *p* < 0.001, partial η^2^ = 0.353). Since participants overall were more likely to select risky decisions (μ = 63.6%; SE = 0.004; one-sample *t* test: *t* = 33.037; *p* < 0.001), we infer that this observed difference in response time is likely due to switching costs (for a detailed account on the voluntary switch cost, see Arrington et al., 2014).

**Figure 3.**
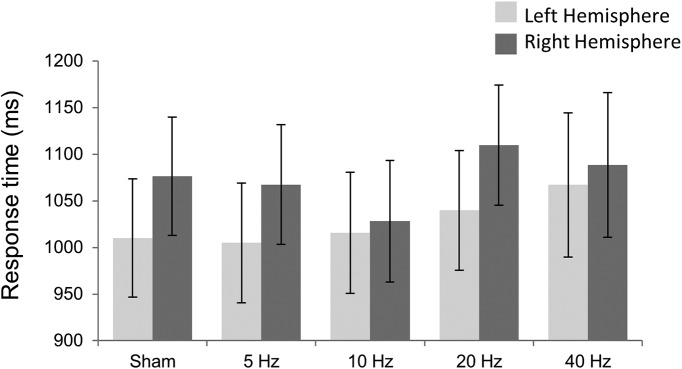
Mean response times for each Hemisphere group across frequency stimulation.

In addition, the mixed ANOVA on response time revealed a main effect of valence (*F*_(1,32)_ = 25.842; *p* < 0.001, partial η^2^ = 0.447), showing slower mean response times in loss blocks (μ = 1085.96 ms) compared to gain blocks (μ = 1015.44 ms). This significant difference may indicate increased deliberation in loss blocks. No other effects on reaction times were significant. See Figure 3 and Table 5 for list of response times for each condition.

**Table 5.** Mean response times associated with each hemisphere group across frequency stimulation

				95% confidence interval	
Hemisphere	Frequency	Mean	SE	Lower bound	Upper bound
Right	Sham	1009.92	63.41	880.75	1139.09
	5 Hz	1004.91	64.29	873.96	1135.86
	10 Hz	1015.71	65.06	883.19	1148.23
	20 Hz	1039.81	64.26	908.91	1170.71
	40 Hz	1067.01	77.55	909.05	1224.98
Left	Sham	1076.12	63.41	946.95	1205.29
	5 Hz	1067.47	64.29	936.52	1198.42
	10 Hz	1028.15	65.06	895.63	1160.66
	20 Hz	1109.68	64.26	978.79	1240.58
	40 Hz	1088.27	77.55	930.30	1246.23

## Discussion

In the attempt to modulate oscillatory activity underling voluntary risky decision making and executive control we applied tACS (sham, 5, 10, 20, and 40 Hz) to the left and right PFC while participants performed a modified risky decision-making task that requires choosing between risky and certain decisions by switching or repeating task sets. The analyses of risky decision making revealed several significant effects, yet the influence of a 20-Hz stimulation on risky decision making was the most robust, surviving Holm-Bonferroni correction. Although frequency specificity has been demonstrated with 20-Hz tACS for motor (Pogosyan et al., 2009; Feurra et al., 2011, Joundi et al., 2012) and sensory functions (Kanai et al., 2008, 2010; Turi et al., 2013), the current experiment is the first to reveal a frequency-specific increase in voluntary risky decision making from 20-Hz tACS.

Within recent years, EEG studies investigating oscillatory activity in gambling tasks have demonstrated a correspondence between frontal β-oscillations (20–35 Hz) and anticipation of probable rewards (Bunzeck et al., 2011), as well as receiving unexpected rewarded feedback (Marco-Pallares et al., 2008; HajiHosseini et al., 2012; HajiHosseini and Holroyd, 2015; Mas-Herrero et al., 2015). Marco-Pallarés et al. (2015) proposed that frontal β-oscillatory activity during gambling paradigms might signify the functional coupling between cortical and subcortical regions such as the ventral striatum, known to be involved in reward processing. This was recently confirmed in an EEG-fMRI study that reported correspondence between mid-frontal β-oscillatory activity and engagement of the fronto-striatal-hippocampal network (Mas-Herrero et al., 2015). This may indicate that 20 Hz of stimulation increased motivation to select risky decisions by indirectly affecting brain regions of the reward system, such as the ventral striatum. Importantly, the ventral striatum is a key subcortical region for risky decision making since the activation of this area predicts risky decision making and increases in activation as rewards become more probable (Knutson and Greer, 2008; Niv et al., 2012). Taken together, we speculate that stimulation of the frontal cortex with 20-Hz tACS may have resulted in a boost in reward-related processes involving the ventral striatum, thus resulting to an increase in voluntary risky decision making. Further support for this claim derives from electrical simulations of the left PFC (F3, EEG 10–20 system) with an extracephalic electrode placed on the shoulder demonstrating modulation of the PFC and deep medial structures (Bai et al., 2014).

It is important to underline that although several effects involving hemisphere of stimulation were statistically significant, these effects should be generalized to the population cautiously since we used a between group design combining random effects (valence, switch condition, and frequency of stimulation) with fixed effects (Hemisphere of stimulation). Importantly, the specific effect of 20-Hz tACS of the left PFC on risky decision making was further confirmed by separate statistical analyses for the left- and right-side stimulation (Table 2). Another potential caveat to the study is that potential after-effects of tACS cannot be ruled out as no simultaneous EEG recording took place. Despite the growing evidence that tACS effects neural oscillatory activity online (Antal et al., 2008; Helfrich et al., 2014a,b; Strüber et al., 2015), it was not possible to control within the current experiment.

The results of the current study seem contradictory to a previous study using tACS on risky decision making (Sela et al., 2012). However, the effect of θ-band tACS in the previous study (Sela et al., 2012) could be due to a modulation of feedback-related adjustments (Cavanagh et al., 2010; Cavanagh et al., 2012; Luft, 2014; Zhang et al., 2014) since the previous tACS paper used the Balloon Analog Risk Task, which measures risk-taking propensity across a cumulative number of responses, as opposed to measuring risky decision making within a single response, as in the current study. A possible explanation for the alternate results may be due to the differences in montage. For instance, a previous study that modulated executive functions, specifically working memory, stimulated both frontal and parietal areas using an F3–P3 montage (Polanía et al., 2012). We suggest that stimulation of the frontal lobe may modulate either a frontal-striatal network associated with voluntary risky decision making (Rao et al., 2008) or a frontal-parietal network in association with voluntary executive control (Orr and Banich, 2014) depending on the placement of the reference electrode (Bai et al., 2014). Whereas the F3–EC (extracephalic) montage used in the current study likely modulates frontal and deep medial structures, an F3–P3 montage likely modulates frontal and parietal structures (Bai et al., 2014). Therefore, modulation of voluntary executive control may require an F3–P3 montage. Some have reported that the ratio of θ- and β-oscillations at resting state can be used to predict risk preferences in individuals (Schutter and van Honk, 2005; Massar et al., 2014). Therefore, it is also plausible that both θ- and β-band stimulation may modulate different cognitive components of the decision-making process within different states and/or contexts. Alternatively, one may suggest that the 20-Hz stimulation could modulate working memory during risky decision making. Some studies suggest that risky decision making is associated with the capacity to maintain and organize information in working memory as an estimation of executive processes (Brevers et al., 2014a,b). Unfortunately, our study design did not allow testing of this hypothesis. However, we think that a modulation of working memory should not affect our results since subjects continued to receive training until their performance became above 95%, as specified in the stimuli and procedure section, thereby eliminating potential confound learning effects and an overload of working memory.

Finally, our findings are consistent with the previous studies demonstrating that laterality (left and right frontal hemisphere) strongly influences the effect of voluntary risky decision making (Knoch et al., 2006; Fecteau et al., 2007a,b; Sela et al., 2012; Cheng and Lee, 2016). Together, these previous studies show that exciting the left and/or inhibiting the right PFC increasing risky decision making and vice versa. This suggests that a 20-Hz stimulation increases cortical excitability of the left frontal area, presumably by entraining the frontal-striatal network. Together these results offer novel insight into the role of β-oscillatory activity in neural mechanisms of risky decision making.

## Synthesis

Reviewing Editor: Philippe Tobler, University of Zurich Decisions are customarily a result of the Reviewing Editor and the peer reviewers coming together and discussing their recommendations until a consensus is reached. When revisions are invited, a fact-based synthesis statement explaining their decision and outlining what is needed to prepare a revision will be listed below. The following reviewer(s) agreed to reveal their identity: Peter Mohr.

In this study, the authors investigate the effect of tACS (applied over the PFC at different frequencies) on risky decision-making and cognitive control. The results show that tACS at 20 Hz has a specific role on risky decision-making, where the effects appear to be driven by tACS applied over the left PFC. The reviewers found merit in the study and noted that the findings were novel and that it is important to study the potential role of tACS neural entrainment on risk-taking behavior and possibly establish a causal link between this type of behavior and neural oscillations. However both of the reviewers also had several concerns that need to be addressed thoroughly in order for the paper to be considered further.

Major concerns

1. Introduction: Both reviewers criticized that the introduction lacks clear hypotheses. What is the exact hypothesis that the authors want to test? They should derive it in a clear and concise way from the literature and design their experiment accordingly. Moreover, the authors should link the PFC not only very broadly to risk-taking behavior but to specific components of risky decision making (e.g., risk or risk attitude - for example, several studies have shown that risk affects lateral prefrontal value signals according to individual risk attitude, which contrasts with pure choice-dependent risk (variance) signals in anterior cingulate cortex). Moreover, please provide the rationale that led the authors to conduct this study in terms of oscillations and the motivation to choose these specific frequencies e.g. Why did the authors go for 5 Hz instead of 6.5 Hz, which seems odd given that they write that 6.5 is what has been commonly observed in risk-tasks. Maybe using 6.5 would have increased the marginal effect observed for 5 Hz?

a. Related point, Introduction: The link between risk-taking and voluntary cognitive control is weak. The authors are certainly aware of the reverse inference problem. Only building on PFC activations, one could link dozens of findings to risk-taking. The authors would have to come up with additional arguments why they think the two concepts are linked.

b. Related point, Introduction: The authors use the terms risk-taking, decision-making under risk and risky decision making quite interchangeably. They should clarify what they mean exactly with these terms.

c. Related point, Introduction: “To manipulate and isolate the degree of regulatory control on choices and risk-taking, [...]” What is the difference between choices and risk-taking in this case?

2. Methods: The way the statistical analysis was carried out needs to be more clearly specified. The authors write: “Other predictors were modelled as fixed effects”. Does it mean that all regressors are treated as fixed effects? If this is the case, the statistical results reported by the authors cannot be generalized to the population, as outliers or just an average trend can easily drive the observed fixed effect. It is appreciated that the authors corrected for multiple comparisons the fixed-effects p-values, however, this does not prevent the issue brought up here. Please spell out in the manuscript the R command lme4 function used to run the hierarchical model in order to clarify how exactly the regression was carried out.

3. Methods: If we understand the task right, the execution of a choice (safe vs. risky) crucially depends on working memory. Did the authors control for differences in working memory ability?

4. Methods: It would be important to know the area affected by the electrode montage used in this study. It is appreciated that the authors used an extra-cephalic return electrode, which presumably favors the focality of the effect. However, this needs to be demonstrated and reported in the manuscript. An example of a recent tACS study where the electric field model of a montage with an extra-cephalic return electrode was used is Moisa et al., 2016 J. Neuroscience.

5. Methods: Participants were paid based on the feedback outcomes of six randomly selected trials. The authors should clarify why this procedure was chosen and that it is incentive compatible. Could it be the case that participants adjust their risk-taking behavior because not only one trial is paid?

6. Discussion: From the results reported here and the way the discussion is formulated, the mechanism that the authors affected with the application 20 Hz tACS remains unclear - was it entrainment of the Beta oscillations that facilitated task performance, or was it enhancement of cortical excitability as reported in the Feurra study. In case of the latter, given that the stimulation lasted 5-10 mins, how did this affect the behavior on the subsequent stimulation of other frequencies? Can we conclude that oscillations are affected?

7. Discussion: It would be nice to read more about how stimulation of the PFC might affect reward processing. What would be the neurobiological mechanism underlying the effect.

Minor comments

1. The abstract is unclear. For instance at the end of the first sentence: “decision making and decision to exert....”, please reformulate this in a more transparent manner. Also in line 10, the authors write “strong effect”, what is strong effect?. Also the last sentence “underlined by beta oscillatory activity”, is a strong statement.... Maybe it is just cortical excitability enhancement (see major point 6, above)

2. Methods: The authors should provide demographics for the participants in each condition separately.

3. Results: It would be great to see a figure with the reaction times next to the %Risk figures

4. Results: Please also provide a table for the RT ANOVA analyses

5. Results: The authors write “highly significant”. We would suggest deleting “highly”.

6. Table 1: Please make clear that p' corresponds to the corrected p-value

7. There are several typos throughout the manuscript. Perhaps a professional language editing would help.
